# Attentional Control and Fear Extinction in Subclinical Fear: An Exploratory Study

**DOI:** 10.3389/fpsyg.2017.01654

**Published:** 2017-09-26

**Authors:** Eduard Forcadell, David Torrents-Rodas, Devi Treen, Miquel A. Fullana, Miquel Tortella-Feliu

**Affiliations:** ^1^Department of Psychiatry and Forensic Medicine, School of Medicine, Universitat Autònoma de Barcelona, Barcelona, Spain; ^2^Department of Mental Health, Parc Taulí Hospital Universitari, Institut d’Investigació i Innovació Parc Taulí I3PT, Universitat Autònoma de Barcelona, Sabadell, Spain; ^3^Specialized Department in Mental Health and Intellectual Disability, Institut Assistència Sanitària (IAS), Institut d’Investigació Biomèdica de Girona, Parc Hospitalari Martí i Julià, Salt, Spain; ^4^Institute of Neuropsychiatry and Addictions, Parc de Salut Mar, Barcelona, Spain; ^5^Anxiety Unit, Institute of Neuropsychiatry and Addictions, Hospital del Mar, CIBERSAM, Barcelona, Spain; ^6^IMIM, Hospital del Mar Medical Research Institute, Barcelona, Spain; ^7^University Research Institute on Health Sciencies (IUNICS), Universitat de les Illes Balears, Mallorca, Spain; ^8^PROMOSAM Red de Investigación en Procesos, Mecanismos y Tratamientos Psicológicos para la Promoción de la Salud Mental, Mallorca, Spain

**Keywords:** attentional control, attentional network functioning, fear extinction, extinction learning, extinction recall, anxiety disorders

## Abstract

Attentional control (AC) and fear extinction learning are known to be involved in pathological anxiety. In this study we explored whether individual differences in non-emotional AC were associated with individual differences in the magnitude and gradient of fear extinction (learning and recall). In 50 individuals with fear of spiders, we collected measures of non-emotional AC by means of self-report and by assessing the functioning of the major attention networks (executive control, orienting, and alerting). The participants then underwent a paradigm assessing fear extinction learning and extinction recall. The two components of the orienting network functioning (costs and benefits) were significantly associated with fear extinction gradient over and above the effects of trait anxiety. Specifically, participants with enhanced orienting costs (i.e., difficulties in disengaging attention from cues not relevant for the task) showed faster extinction learning, while those with enhanced orienting benefits (i.e., attention facilitated by valid cues) exhibited faster extinction recall as measured by fear-potentiated startle and Unconditioned Stimulus expectancies, respectively. Our findings suggest that, in non-emotional conditions, the orienting component of attention may be predictive of fear extinction. They also show that the use of fear extinction gradients and the exploration of individual differences in non-emotional AC (using performance-based measures of attentional network functioning) can provide a better understanding of individual differences in fear learning. Our findings also may help to understand differences in exposure therapy outcomes.

## Introduction

Several attentional and learning processes have been found to play a major role in pathological anxiety (i.e., anxiety disorders). Recent research suggests that attentional control (AC) and fear extinction learning feature prominently among such processes (e.g., Bar-Haim et al., 2007; Cisler and Koster, 2010; Eysenck and Derakshan, 2011; Milad and Quirk, 2012; Heeren et al., 2013; VanElzakker et al., 2014; Duits et al., 2015; Hadwin et al., 2016). In this study, we explore the possible association between these two processes.

Attentional control is a construct that defines our ability to regulate attention allocation, including our ability to maintain sustained attention, ignore distractors, and shift attention between tasks (Derryberry and Reed, 2002). Deficient AC has been found to characterize both clinical (Olatunji et al., 2011) and subclinical anxiety (e.g., Fajkowska and Derryberry, 2010; Sportel et al., 2013). Such deficits may also account for the attention bias to threat commonly observed in anxious individuals (Hadwin et al., 2016). Moreover, such deficits are associated with reduced ability to regulate emotion (Fajkowska and Derryberry, 2010; Armstrong et al., 2011; Tortella-Feliu et al., 2014; Hsu et al., 2015; Morillas-Romero et al., 2015; O’Bryan et al., 2017). AC can be assessed under emotional conditions (emotional AC, e.g., Barry et al., 2013) or neutral conditions (non-emotional AC, e.g., Derryberry and Reed, 2002; Pacheco-Unguetti et al., 2011; Richey et al., 2016).

Fear extinction learning refers to the decrease in fear following non-reinforced exposure to a feared conditioned stimulus (CS) and is typically investigated in humans within a differential fear learning paradigm preceded by a conditioning (i.e., acquisition) phase. The test of how fear extinction learning is retrieved after re-exposure to the extinguished CS is usually called extinction recall. Deficient fear extinction learning (Duits et al., 2015) or extinction recall (Graham and Milad, 2011; Milad and Quirk, 2012) could be a marker of anxiety disorders (e.g., Graham and Milad, 2011). Importantly, fear extinction is a form of emotion regulation (Hartley and Phelps, 2010) and there is evidence that similar neurobiological mechanisms (i.e., hipoactivity of the prefrontal cortex) may be involved in fear extinction, reduced emotion regulation capabilities, and low AC in non-emotional conditions (Bishop, 2007, 2008, 2009; Hartley and Phelps, 2010; Milad and Quirk, 2012; Ochsner et al., 2012; Shiba et al., 2016; Ball et al., 2017). Therefore, individual differences in AC under non-emotional conditions may be associated with individual differences in fear extinction, although this has not been investigated so far, as far as we are aware.

Moreover, fear extinction learning procedures are considered experimental models for exposure therapy (Craske et al., 2014: Pittig et al., 2016) and both fear extinction learning (e.g., Waters and Pine, 2016; Ball et al., 2017; Forcadell et al., 2017) and attentional functioning (e.g., Barry et al., 2015a) may be associated with the outcomes of exposure therapy, and constitute putative targets for improving such outcomes (Bar-Haim, 2010; Craske et al., 2012, 2014; Barry et al., 2015b; Mogg and Bradley, 2016; Pittig et al., 2016). A better understanding of the association between AC and fear extinction may therefore have important therapeutic implications.

The role of attention in fear learning has been a topic of research for years (e.g., Mackintosh, 1975; Wagner, 1981; Le Pelley et al., 2016). Most studies have focused on how attention allocation changes during or after acquisition (e.g., Beaver et al., 2005; Koster et al., 2005) or extinction (e.g., Robbins, 1990; Van Damme et al., 2006; Barry et al., 2016b) affect the magnitude of learning. Moreover, attentional biases to threat in anxiety could reflect a much broader dysregulation of AC (Bishop, 2009; Moriya and Tanno, 2009; Pacheco-Unguetti et al., 2011). The use of non-emotional stimuli allows to isolate potential general attention deficits beyond those observed when individuals face emotional materials (see further below). A few recent studies have focused on how baseline individual differences in attention predict the magnitude or gradient (“speed”) of fear extinction learning (Waters and Kershaw, 2015; Barry et al., 2016a, 2017).

The study by Waters and Kershaw (2015) belongs to the research tradition that focuses on analyzing how attentional bias to threat-related information is associated with increased anxiety (valence-specific models) (for a review see Heeren et al., 2013; Hadwin et al., 2016). Waters and Kershaw (2015) found that clinically anxious children who showed attention to threat in a visual probe task exhibited greater fear extinction learning than those who avoided threat.

The studies by Barry and colleagues (Barry et al., 2016a, 2017) represent a second research tradition that has explored how deficient AC is associated with anxiety vulnerability, and more specifically with cognitive and inhibitory control impairments observed in anxious individuals. In two separate studies in healthy participants, these authors investigated how emotional AC, as measured by self-report (Barry et al., 2013), was associated with fear extinction learning (Barry et al., 2016a, 2017). In the first study, participants were confronted with a perceptually similar stimulus presented after extinction of the original CS, and it was observed that higher emotional AC was associated with faster extinction learning and greater return of fear (Barry et al., 2016a). In the second study, during extinction learning participants were confronted with a similar stimulus as during acquisition, and were instructed to attend toward the common features between the acquisition and extinction stimuli, or toward the unique features of the extinction stimulus. For participants who, during extinction, were instructed to attend toward the unique features of the extinction stimulus, lower emotional AC tended to be associated with a greater return of fear (Barry et al., 2017). The authors suggested that those with low emotional AC may have been unable to shift attention to other features of the extinction stimulus, which may have facilitated the return of fear when confronted with a perceptually similar stimulus.

In the studies mentioned above, individual differences in attention functioning were investigated using emotional conditions. We share the view of Heeren et al. (2015b, p. 136) that the focus on emotional materials has “neglected the empirical exploration of basic attentional deficits from non-emotional material,” and precludes the assessment of general attentional abilities that may be relevant to several clinical phenomena (see also Snyder et al., 2015). Furthermore, in the two studies investigating the association between AC and extinction learning, AC was assessed by self-report (Barry et al., 2016a, 2017). The use of performance-based tasks (see below) may provide important information on the role of different attentional networks beyond general AC (see Heeren et al., 2015a; Heeren and McNally, 2016).

According to the *attention system model* (Posner and Petersen, 1990; Posner and Rothbart, 2007), attention consists of three major networks, which can be assessed separately: *executive control*, *orienting*, and *alerting* (see Posner et al., 2007 for a review). The *executive control* network is specialized in conflict resolution and voluntary control of attention while resisting distraction by other competing stimuli. While the executive control network has traditionally been equated to AC, some authors have recently expanded the definition of AC to include the orienting and alerting networks (e.g., Heeren and McNally, 2016). The *orienting network* is involved in attention engagement to new stimuli and attention disengagement from the current focus. Finally, the *alerting network* is devoted to maintaining adequate sensitivity to perceive and process stimuli. The functioning of these three attentional networks when facing non-emotional cues has been related to anxiety and emotion regulation. For example, reduced efficiency of the executive control and orienting networks has been associated with high trait and clinical anxiety (Moriya and Tanno, 2009; Pacheco-Unguetti et al., 2010; Pacheco-Unguetti et al., 2011; Heeren et al., 2015b; Heeren and McNally, 2016), and faster spontaneous emotion regulation (Morillas-Romero et al., 2015). Finally, increased efficiency of the alerting network has been associated with state anxiety (Pacheco-Unguetti et al., 2010).

In this present study, we explore whether individual differences in non-emotional AC (defined as a multifaceted construct including executive control, orienting, and alerting attentional networks), are associated with individual differences in fear extinction (learning and recall) in a sample of subclinical phobic participants (individuals with moderate to strong fear of spiders). The use of subclinical samples is a valid strategy for studying anxiety-related processes, can be generalized to individuals with an anxiety diagnosis (Stopa and Clark, 2001; Abramowitz et al., 2014) and also has some advantages (e.g., avoid comorbidity, medications or the impact from previous treatments) compared to clinical samples. Furthermore, previous studies exploring the association between attentional bias to threat and fear extinction (Waters and Kershaw, 2015) and between fear learning and treatment outcome (Waters and Pine, 2016) included children with specific phobias, but to the best of our knowledge, ours is the first study exploring the role of non-emotional attention and its association with fear extinction in (subclinical) adult phobic individuals using “truly” non-emotional stimuli.

We used self-report and performance-based measures of AC under non-emotional conditions. Fear extinction was assessed using three different measures: Unconditioned Stimulus (US) expectancies, Skin Conductance Response (SCR), and Fear-Potentiated Startle (FPS). Given the well-established association between trait anxiety and AC (e.g., Pacheco-Unguetti et al., 2010, 2011; Sportel et al., 2011), and between trait anxiety and fear extinction (Sehlmeyer et al., 2011; Gazendam et al., 2013; Haaker et al., 2015), we tested the magnitude of these associations after controlling for trait anxiety.

## Materials and Methods

### Participants

**FIGURE 1:**
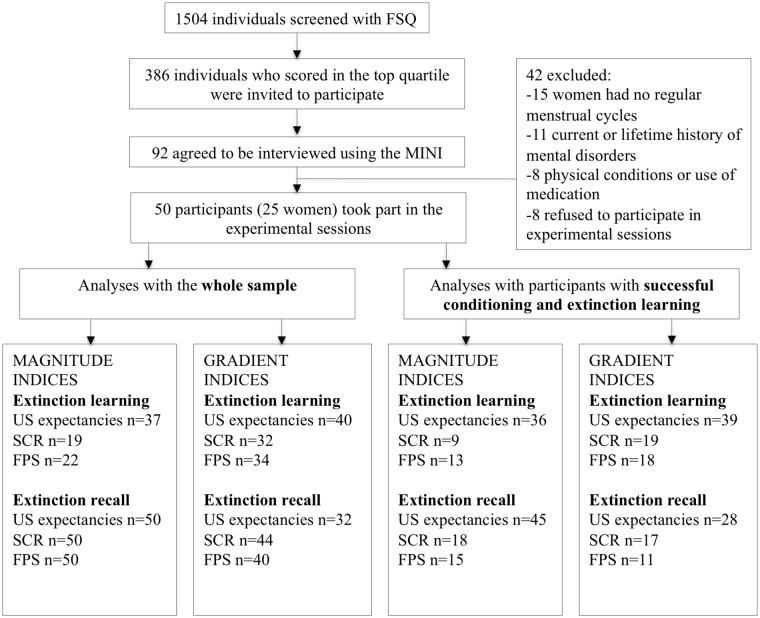
Diagram of the sample selection procedure and participants included in each analysis. FSQ, Fear of Spiders Questionnaire; MINI, Mini International Neuropsychiatric Interview; US, Unconditioned stimulus; SCR, Skin Conductance Response; FPS, Fear-Potentiated startle.

We selected individuals with moderate to strong fear of spiders, as assessed by a dimensional instrument. Participants were recruited by advertisement to participate in a study on “physiological responses to anxiety” (see participants flow chart in **Figure 1**). Initially, 1504 individuals were screened with the validated Spanish version (Forcadell et al., 2014) of the *Fear of Spiders Questionnaire* (FSQ; Szymanski and O’Donohue, 1995) via a secure web system. In the online stage we used online forms with encryption technology that guaranteed the privacy of the participants. The information could only be processed by a person with access to the matrix and passwords. Participants who scored in the top quartile of the study distribution (FSQ ≥ 33; *n* = 386) were invited to participate. Of those, 92 agreed to be interviewed by a doctoral-level clinical psychologist using the Mini International Neuropsychiatric Interview (MINI; Sheehan et al., 1998).

Exclusion criteria were: (a) current or lifetime history of mental disorders other than specific phobia (animal type, spiders), as determined by the MINI, supplemented with the specific phobia section of the Structured Clinical Interview for DSM (SCID; First et al., 2002); (b) use of medication/illicit drugs or medical problems that could interfere with study performance or interpretation; (c) alcohol abuse; (d) pregnancy; (e) not being Spanish-speaker. Female participants had regular menstrual cycles (as per self-report), had not used oral contraceptives or hormone replacement therapy during the previous 3 months (as per self-report), and participated in the study during their early follicular phase (days 3–8 of a 28–30-day cycle) to avoid possible confounding by sex hormones in fear extinction (Milad et al., 2006; Merz et al., 2012; Pineles et al., 2016). All participants were tested between 5 and 8 PM.

The final sample consisted of 50 participants with moderate to strong fear of spiders (*M_FSQ_* = 58.98, *SD* = 17.94; *M*_age_ = 21.50 years, *SD* = 2.93; 25 women). The number of participants included in each analysis is reported in **Figure 1**. Participants gave written informed consent to take part in the study, which was approved by the corresponding institution’s Clinical Research Ethics Committee. Participants were paid €25.

### Procedure Overview

**FIGURE 2:**
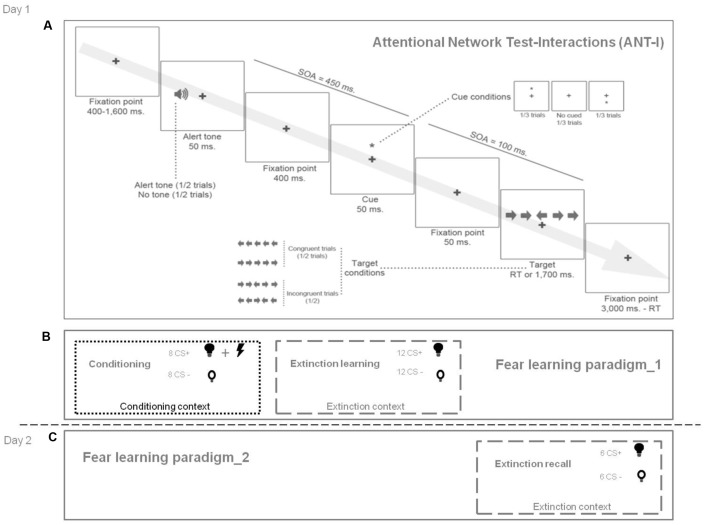
Summary of experimental design. **(A)** Attentional Network Test-Interactions (ANT-I): Trials began with a fixation point (400–1600 ms). Then, in half of the trials, an alerting tone is presented (50 ms). In two-thirds of the trials, this is followed after 400 ms by an asterisk as an orienting signal (50 ms) either above or below the fixation point (cued trials). No asterisk is presented in the remaining third of the trials (uncued condition). Then, the asterisk disappears leaving only the fixation point. After 50 ms, an arrow flanked by four distractor arrows (two on each side) is presented. The distractors point either in the same direction as the arrow target (i.e., congruent trials) or in the opposite direction (i.e., incongruent trials), and in the same position as the orienting cue (i.e., valid trials) or at the opposite location (i.e., invalid trials). Participants have to indicate the direction in which the target was pointing (right or left). After the response, the fixation point is presented for up to 3000 ms. **(B)** Fear learning paradigm, day 1 (conditioning and extinction learning). A conditioning phase was followed by an extinction learning phase. The visual contexts were photographs of two different rooms (i.e., conditioning context and extinction context). A lamp switched on to one of two different colors (blue or yellow), which were the CS+ and the CS–. Participants were not instructed about the CS-US contingency. During conditioning, the CS+ co-terminated with an electric shock (US). During extinction learning (immediately after conditioning), the CS+ was not followed by the US. The CS– was never followed by the US. **(C)** Fear learning paradigm, day 2 (extinction recall). This phase was identical to extinction learning on day 1. The extinguished CS+ and the CS- were presented again in the extinction context. SOA, stimulus onset asynchrony; RT, reaction time; CS+, conditioned stimulus associated with the unconditioned stimulus during the conditioning phase; CS–, conditioned stimulus never associated with the unconditioned stimulus; US, unconditioned stimulus.

Participants took part in two experimental sessions on 2 consecutive days. On day 1, they completed (a) a self-report measure of AC (Attentional Control Scale; ACS; Derryberry and Reed, 2002), (b) the trait version of the State-Trait Anxiety Inventory (STAI-T; Spielberger et al., 1970), (c) a task assessing attentional network functioning (Attentional Network Test-Interactions task, ANT-I, Callejas et al., 2004) (see below and **Figure 2A**), and they underwent the first part of the fear learning paradigm (conditioning and extinction learning) (see below and **Figure 2B**). On day 2, they participated in the second part of the fear learning paradigm (extinction recall) (**Figure 2C**). Psychophysiological responses were recorded continually during the fear learning paradigm (see below).

### Self-report Measures

#### Attentional Control

We used the Spanish version (Pacheco-Unguetti et al., 2011) of the Attentional Control Scale (ACS; Derryberry and Reed, 2002) to measure individual differences in non-emotional AC (e.g., “My concentration is good even if there is music in the room around me,” “It is easy for me to read or write while I am also talking on the phone”). The scale consists of 20 4-point items (1 = *Almost never*; 4 = *Always*), with higher scores indicating higher AC. The scale is divided into two subscales: focusing (i.e., ability to intentionally hold attentional focus) and shifting (i.e., ability to intentionally shift attentional focus). In line with Ólafsson et al. (2011), item 9 was excluded when calculating the total score. Cronbach’s alpha for the ACS was 0.70.

#### Trait Anxiety

We used the Spanish version (Spielberger et al., 1982) of the STAI-trait (STAI-T; Spielberger et al., 1970) to measure trait anxiety (e.g., “I worry too much over something that really does not matter,” “I am content”). It consists of 20 4-point items (1 = *Almost never*; 4 = *Almost always*). Scores range from 0 to 60 in the Spanish version of the STAI-T, with higher scores indicating higher trait anxiety. Cronbach’s alpha for the STAI-T was 0.87.

### Performance-Based Measures

#### Attentional Network Functioning

We used the Attentional Network Test for Interactions (ANT-I; Callejas et al., 2004), a modified version of the Attentional Network Test (Fan et al., 2002), to assess the functioning of the three major attentional networks (executive control, orienting and alerting), and the two components of orienting, namely costs (i.e., disengagement of attention from invalid cues), and benefits (i.e., facilitated orientation from valid cues). The task consisted of four blocks of 48 trials. On each trial, a non-emotional cue (an alerting tone and/or an asterisk) preceded an arrow flanked by other distractor arrows (see **Figure 2A**). Participants had to indicate the direction of the target arrow by pressing one of two keys as quickly and accurately as possible (we measured reaction time and error rate; see below). Following previous research (Callejas et al., 2004; Pacheco-Unguetti et al., 2010, 2011) we computed an efficiency index for each attentional network, and for the two components of orienting (see Supplementary Methods), where higher scores indicated enhanced functioning of the network (except for executive control and orienting costs, where higher scores indicated diminished functioning).

### Fear Learning Paradigm

We adapted the paradigm developed by Milad et al. (2005) which assesses conditioning, extinction learning, and extinction recall separately. The original task used Skin Conductance Response (SCR) as the only measure of conditioned fear, and we added two other measures (Unconditioned Stimulus [US] expectancies and Fear-Potentiated Startle [FPS]). Briefly, the visual contexts were photographs of two different rooms (conditioning context, CX+; extinction context, CX-) containing a lamp that switched on to one of two different colors (blue or yellow), which were the CS+ and the CS- (CS not paired with the US). Contexts and CSs were displayed on a computer monitor in front of the participant. On day 1, a conditioning phase (in CX+) was followed by an extinction learning phase (in CX-). During conditioning, the CS+ co-terminated with an electric shock (US). The US was individually adjusted before the experiment (day 1), presenting shocks of gradually increasing intensity until a ‘definitely annoying but not painful’ shock was selected [*M*_shocklevel_ = 4.9 milliamperes (mA), *SD* = 3.3]. Participants were not instructed about the CS-US contingency. During extinction learning (immediately after conditioning), the CS+ was not followed by the US. The CS– was never followed by the US. The extinction learning phase was divided in two equal parts by a 1-min pause (early and late extinction learning). Day 2 consisted of an extinction recall phase (in CX-). During day 2, the CS+ and the CS– were never followed by the US. The US was not recalibrated during day 2.

Each trial of the experiment started with presentation of the context for 10, 12, or 14 s. Then the CS was presented (i.e., the lamp switched on) for 8 s, and a startle probe (50 ms duration, 100 dB) was delivered 7 s after CS onset. Between trials, a fixation cross was shown for 1 s. In one third of the trials (noise-alone trials, NA), no CS was presented, and instead the context was present for 8 more seconds; the startle probe was presented at second 7 of this extra time. The inter-probe interval varied between 18, 20, and 22 s. Eight trials of each type (CS+, CS–, and NA) were presented during conditioning, and six trials of each type were presented during each of the remaining phases (early and late extinction learning and extinction recall). Trial order was randomized across participants in blocks of nine trials (three of each type), with the restriction that no more than two trials of the same type occurred consecutively. Assignment of the photographs of the rooms to the conditioning and the extinction contexts, and of the CS+ and the CS–, was counterbalanced across participants.

SCR, FPS, and US expectancy ratings were calculated for each trial type. The SCR signal was sampled at a rate of 125 Hz. SCR magnitudes were computed in microsiemens (μS) as the difference between the maximum SCR value and the value at response onset, occurring 1–7 s after CS onset. Trials in which no response was detected or with a response magnitude of <0.02 μS were considered non-response trials (see Dunsmoor et al., 2009), and trials showing interference or excessive baseline activity (1.3%) were rejected after visual inspection. To normalize the distribution of the SCR data, we applied a square root transformation.

Startle amplitudes were computed in microvolts (μV) as the difference between the EMG value at response peak and the average EMG during the 50 ms preceding the probe. If no response was detected in a given trial, the amplitude was scored as 0 μV. To be considered a valid response, elevations in the EMG recording had to start between 20 and 100 ms, and their peak had to occur between 20 and 150 ms after the probe. After visual inspection, trials with excessive noise (3.2%) were rejected. Raw data were transformed into T-scores to control for differences in reactivity. Scorers of SCR and FPS data were blinded to the stimuli presented.

Regarding US expectancy ratings, for each trial participants were told to try to predict whether the shock would occur in the following seconds each time the lamps in the rooms turned blue or yellow. They had to answer as quickly as possible by clicking on the scale from 0 (no shock) to 10 (shock) displayed at the bottom of the screen (see Supplementary Materials-Methods for further information).

### Fear Extinction Indices

For each participant, we calculated an index based on the magnitude (amount of learning) and an index based on the gradient (slope of change, i.e., “speed”) for both extinction learning and extinction recall.

#### Magnitude-Based Indices

Based on previous research (Rabinak et al., 2013, 2014; Garfinkel et al., 2014; Pineles et al., 2016), for each participant and each measure (US expectancies, SCR and FPS) we calculated an index expressing the “amount of learning,” reflecting differences between CS+ and CS- during extinction learning and extinction recall.

The extinction learning index was calculated according to Pineles et al. (2016): Extinction learning = 100 – ([Mean [CS+]-[CS-] during the last two trials of early extinction/Mean [CS+]-[CS-] during the first two trials of early extinction × 100]). Since the extinction learning index was calculated as a percentage, higher scores indicated enhanced extinction learning (i.e., less discrimination between CS+ and CS-).

The extinction recall index was calculated, based on Rabinak et al. (2013), as the mean of (CS+)-(CS-) in the first two extinction recall trials. Lower scores indicated enhanced extinction recall.

#### Gradient-Based Indices

In line with Barry et al. (2016a, 2017), we computed the slope of change (i.e., gradient) across extinction learning and extinction recall trials using the area under the curve with respect to the decrease in US expectancies, SCR, and FPS scores. For each participant we calculated the percentage change in the difference between CS+ and CS- in each trial during extinction learning and recall relative to the first extinction trial. We used the percentage change between the first and last extinction trials as the baseline to account for individual differences in the intercept of the extinction curves. Lower scores in the gradient-based indices indicated faster fear extinction learning and extinction recall.

### Statistical Analyses

In manipulation checks, we examined main effects and interactions between networks and reaction time in the ANT-I using a factorial mixed ANOVA with executive control (congruent and incongruent), orienting (valid, invalid, uncued), and alerting (no alerting, alerting tone) as within-subject factors, and reaction time as the dependent variable. We also studied the association between trait anxiety and AC variables using Pearson bivariate correlations. We also studied the performance of the sample during the fear learning paradigm using repeated-measures ANOVA for each phase (conditioning, extinction learning and extinction recall) and for each measure (US expectancies, SCR and FPS), with CS type (CS+ vs. CS-) and Block as within-subject factors. We averaged SCR and FPS responses over two consecutive trials of the same type (blocks), and applied Greenhouse–Geisser corrections for main effects and interactions involving repeated measures. For indices calculated as a percentage, extreme values (>150% or <-150%) were excluded.

We used Pearson bivariate correlation analyses to test for association between AC with fear extinction indices. Following recent guidelines for the analyses of fear learning data in humans (Lonsdorf et al., 2017), we performed those analyses with the whole sample. Since extinction learning can theoretically only occur if there is conditioning, and extinction recall can occur only if there is extinction learning, we repeated our extinction learning analyses using only those participants showing successful conditioning, and our extinction recall analyses using only those participants showing successful extinction learning. The criteria for establishing successful fear conditioning/extinction learning were based on Schiller et al. (2012): the differential SCR to the CS+ and CS- by the end of the conditioning phase (mean of second half of the conditioning trials) had to be in the right direction (i.e., CS+ > CS-) and >0.1 μs for SCR, >1 μV for FPS, or >1 point for US expectancies. Similarly, the criteria for establishing successful extinction learning were: differential SCR to the CS+ and CS- by the end of the late extinction learning phase (last trial) had to be ≤0.1 μs for SCR, ≤1 μV for FPS, or ≤1 for US expectancies.

For associations that were significant at *p* < 0.10, we conducted hierarchical regression analyses using AC as the independent variables and fear extinction indices as the dependent variables. For these analyses, trait anxiety was entered in Step 1, and the AC variables were entered independently in Step 2.

Finally, we conducted additional analyses using an alternative method to calculate indices based on fear extinction gradients (see Barry et al., 2016a,b).

## Results

### Manipulation Checks

Our manipulation checks on the ANT-I confirmed (see Supplementary Table S1 in Supplementary Results) the typically observed pattern for this task (i.e., reaction times were significantly shorter in: (i) trials including an alerting tone than in trials without this tone, (ii) trials including an orienting signal, and (iii) trials where distractors pointed in the same direction as the arrow target) (e.g., Callejas et al., 2004; Pacheco-Unguetti et al., 2010, 2011). Also consistent with previous literature, trait anxiety was significantly and negatively correlated with AC (see Supplementary Results). Regarding the associations between performance-based and self-reported AC, the overall AC scale was only significantly correlated with performance-based executive control (*r* = -0.438; *p* = 0.001), with a higher self-reported AC indicating a lower interference (i.e., greater executive control). The focusing AC subscale was positively associated with interference (i.e., lower executive control) (*r* = 0.323; *p* = 0.022). No significant correlations were found between self-reported AC and the orienting and alerting networks functioning. All the correlations are depicted in Supplementary Materials, Supplementary Table S2.

For all measures (US expectancies, SCR, FPS), we found evidence of successful conditioning (i.e., higher response to the CS+ than the CS- in the last block of conditioning), which allowed us to investigate fear extinction learning. We also found evidence of extinction learning (i.e., similar response to the CS+ and CS- in the last block of extinction) for all measures (US expectancies, SCR, FPS), which allowed us to investigate extinction recall. For further details, see Supplementary Results.

### Correlational Analyses

#### Relationship between AC and Fear Extinction Magnitude-Based Indices

None of the AC variables investigated was significantly correlated with fear extinction learning or recall, as measured by the magnitude-based indices (**Table 1**).

**Table 1:** Bivariate correlation between attentional control and fear extinction **magnitude**-based indices (results for the whole group and for participants with successful fear conditioning and extinction learning).

	AC total	AC focusing	AC shifting	Executive control	Orienting	Orienting-costs	Orienting-benefits	Alerting
**Extinction learning magnitude**
*Whole group*								
US expectancies *n* = 37	-0.037 (*p* = 0.828)	-0.035 (*p* = 0.838)	-0.026 (*p* = 0.878)	-0.007 (*p* = 0.967)	0.069 (*p* = 0.683)	0.045 (*p* = 0.789)	0.042 (*p* = 0.804)	-0.022 (*p* = 0.898)
SCR *n* = 19	0.104 (*p* = 0.671)	0.154 (*p* = 0.529)	0.035 (*p* = 0.888)	-0.147 (*p* = 0.549)	-0.055 (*p* = 0.824)	0.081 (*p* = 0.742)	-0.133 (*p* = 0.587)	-0.047 (*p* = 0.849)
FPS *n* = 22	0.237 (*p* = 0.289)	0.265 (*p* = 0.234)	0.203 (*p* = 0.365)	-0.282 (*p* = 0.204)	-0.312 (*p* = 0.157)	-0.137 (*p* = 0.543)	-0.253 (*p* = 0.255)	-0.100 (*p* = 0.658)
*Successful conditioning and extinction learning*
US expectancies *n* = 36	-0.009 (*p* = 0.957)	-0.016 (*p* = 0.926)	-0.014 (*p* = 0.936)	-0.036 (*p* = 0.836)	0.041 (*p* = 0.813)	0.053 (*p* = 0.761)	0.000 (*p* = 0.999)	-0.050 (*p* = 0.773)
SCR *n* = 9	0.172 (*p* = 0.657)	0.292 (*p* = 0.447)	0.035 (*p* = 0.929)	-0.477 (*p* = 0.194)	0.417 (*p* = 0.264)	0.171 (*p* = 0.660)	0.445 (*p* = 0.230)	0.361 (*p* = 0.340)
FPS *n* = 13	-0.030 (*p* = 0.922)	0.101 (*p* = 0.743)	-0.042 (*p* = 0.890)	0.013 (*p* = 0.965)	-0.097 (*p* = 0.752)	-0.045 (*p* = 0.885)	-0.077 (*p* = 0.802)	0.045 (*p* = 0.885)
**Extinction recall magnitude**
*Whole group*								
US expectancies *n* = 50	-0.185 (*p* = 0.198)	-0.275 (*p* = 0.053)	-0.088 (*p* = 0.545)	0.222 (*p* = 0.122)	0.103 (*p* = 0.475)	0.097 (*p* = 0.501)	0.028 (*p* = 0.848)	-0.230 (*p* = 0.109)
SCR *n* = 50	-0.051 (*p* = 0.723)	-0.210 (*p* = 0.144)	0.126 (*p* = 0.384)	0.221 (*p* = 0.123)	0.088 (*p* = 0.545)	0.190 (*p* = 0.187)	-0.080 (*p* = 0.579)	-0.203 (*p* = 0.158)
FPS *n* = 50	0.049 (*p* = 0.737)	0.111 (*p* = 0.444)	-0.002 (*p* = 0.987)	0.032 (*p* = 0.823)	0.040 (*p* = 0.784)	-0.079 (*p* = 0.585)	0.123 (*p* = 0.393)	0.043 (*p* = 0.765)
*Successful conditioning and extinction learning*
US expectancies *n* = 45	-0.202 (*p* = 0.184)	-0.278 (*p* = 0.064)	-0.099 (*p* = 0.517)	0.224 (*p* = 0.139)	0.157 (*p* = 0.304)	0.113 (*p* = 0.458)	0.072 (*p* = 0.640)	-0.212 (*p* = 0.163)
SCR *n* = 18	0.134 (*p* = 0.596)	-0.158 (*p* = 0.532)	0.349 (*p* = 0.156)	-0.021 (*p* = 0.934)	0.305 (*p* = 0.218)	0.463 (*p* = 0.053)	-0.014 (*p* = 0.957)	-0.317 (*p* = 0.200)
FPS *n* = 15	0.164 (*p* = 0.560)	0.253 (*p* = 0.363)	0.102 (*p* = 0.716)	-0.001 (*p* = 0.996)	0.144 (*p* = 0.609)	-0.193 (*p* = 0.491)	0.497 (*p* = 0.060)	-0.206 (*p* = 0.461)


*US, unconditioned stimulus; SCR, skin conductance response; FPS, fear-potentiated startle; AC, attentional control*.

#### Relationship between AC and Fear Extinction Gradient-Based Indices

The two orienting network components (costs and benefits) showed a significant negative association with fear extinction (see **Table 2**). For the whole sample, orienting benefits (i.e., facilitated orientation) were inversely correlated with our gradient-based index of extinction recall, as measured with US expectancies (*r* = -0.358; *p* = 0.044), indicating that enhanced facilitated orientation was associated with faster extinction recall (see **Table 2**). When only those participants with successful conditioning and extinction learning were included in the analyses, orienting costs were inversely correlated with our gradient-based index of extinction learning using FPS (*r* = -0.493; *p* = 0.038), indicating that greater difficulty in disengaging attention from invalid cues was associated with faster extinction learning. Moreover, orienting benefits were inversely correlated again with our gradient-based index of extinction recall, as measured by US expectancies (*r* = -0.424; *p* = 0.025). We found no other significant associations between AC and fear extinction learning or recall.

**Table 2:** Bivariate correlation between attentional control and fear extinction **gradient**-based indices (results for the whole group and for participants with successful fear conditioning and extinction learning).

	AC total	AC focusing	AC shifting	Executive control	Orienting	Orienting-costs	Orienting-benefits	Alerting
**Extinction learning gradient**
*Whole group*								
US expectancies *n* = 40	0.109 (*p* = 0.505)	0.136 (*p* = 0.401)	0.089 (*p* = 0.583)	0.056 (*p* = 0.730)	-0.005 (*p* = 0.974)	-0.121 (*p* = 0.457)	0.111 (*p* = 0.495)	0.223 (*p* = 0.167)
SCR *n* = 32	0.260 (*p* = 0.151)	0.313 (*p* = 0.081)	0.030 (*p* = 0.872)	0.178 (*p* = 0.330)	0.054 (*p* = 0.767)	0.028 (*p* = 0.880)	0.037 (*p* = 0.842)	-0.094 (*p* = 0.607)
FPS *n* = 34	0.376 (*p* = 0.129)	0.321 (*p* = 0.064)	0.256 (*p* = 0.143)	-0.032 (*p* = 0.858)	0.051 (*p* = 0.776)	-0.082 (*p* = 0.644)	0.137 (*p* = 0.441)	0.273 (*p* = 0.118)
*Successful conditioning and extinction learning*
US expectancies *n* = 39	0.115 (*p* = 0.486)	0.140 (*p* = 0.396)	0.094 (*p* = 0.571)	0.051 (*p* = 0.757)	-0.012 (*p* = 0.944)	-0.121 (*p* = 0.464)	0.105 (*p* = 0.524)	0.219 (*p* = 0.180)
SCR *n* = 19	-0.066 (*p* = 0.788)	-0.081 (*p* = 0.742)	-0.112 (*p* = 0.647)	0.453 (*p* = 0.052)	0.203 (*p* = 0.405)	-0.060 (*p* = 0.808)	0.278 (*p* = 0.249)	0.144 (*p* = 0.558)
FPS *n* = 18	0.297 (*p* = 0.232)	0.231 (*p* = 0.356)	0.223 (*p* = 0.373)	-0.122 (*p* = 0.628)	-0.408 (*p* = 0.093)	**-0.493 (*p* = 0.038)**	0.098 (*p* = 0.698)	0.432 (*p* = 0.073)
**Extinction recall gradient**
*Whole group*								
US expectancies *n* = 32	-0.149 (*p* = 0.417)	-0.094 (*p* = 0.609)	-0.183 (*p* = 0.315)	-0.063 (*p* = 0.733)	-0.300 (*p* = 0.095)	0.014 (*p* = 0.938)	**-0.358 (*p* = 0.044)**	-0.221 (*p* = 0.223)
SCR *n* = 44	-0.153 (*p* = 0.322)	-0.092 (*p* = 0.551)	-0.171 (*p* = 0.267)	0.153 (*p* = 0.320)	-0.095 (*p* = 0.541)	0.086 (*p* = 0.577)	-0.192 (*p* = 0.212)	-0.070 (*p* = 0.651)
FPS *n* = 40	0.220 (*p* = 0.173)	0.012 (*p* = 0.944)	0.347 (*p* = 0.228)	-0.146 (*p* = 0.369)	-0.067 (*p* = 0.681)	0.041 (*p* = 0.801)	-0.121 (*p* = 0.459)	-0.264 (*p* = 0.099)
*Successful conditioning and extinction learning*
US expectancies *n* = 28	-0.217 (*p* = 0.266)	-0.117 (*p* = 0.554)	-0.275 (*p* = 0.157)	-0.080 (*p* = 0.687)	-0.359 (*p* = 0.061)	-0.012 (*p* = 0.951)	**-0.424 (*p* = 0.025)**	-0.178 (*p* = 0.366)
SCR *n* = 17	0.095 (*p* = 0.716)	0.066 (*p* = 0.800)	0.026 (*p* = 0.921)	0.204 (*p* = 0.433)	-0.213 (*p* = 0.411)	-0.028 (*p* = 0.915)	-0.250 (*p* = 0.333)	0.251 (*p* = 0.332)
FPS *n* = 11	0.192 (*p* = 0.571)	0.171 (*p* = 0.614)	0.268 (*p* = 0.426)	-0.564 (*p* = 0.071)	-0.283 (*p* = 0.400)	-0.283 (*p* = 0.400)	0.092 (*p* = 0.787)	0.010 (*p* = 0.976)


*US, unconditioned stimulus; SCR, skin conductance response; FPS, fear-potentiated startle; AC, attentional control. Significant values in bold*.

Scatter plots for the main findings can be found in the Supplementary Materials (Supplementary Figure S1).

#### Predictive Power of AC on Magnitude and Gradient of Fear Extinction

Results from the hierarchical regression analyses (**Table 3**) showed that, after controlling for trait anxiety, orienting costs were a significant predictor of extinction learning gradient (as measured by FPS), accounting for 27.4% of its variance (*F*[2,15] = 4.91, *p* = 0.023, *R^2^_Adjusted_* = 0.27). In other words, greater difficulty in disengaging attention from invalid neutral cues predicted faster extinction learning beyond what is attributable to trait anxiety.

**Table 3:** Summary of hierarchical multiple regression analyses predicting fear extinction.

	Δ*R*^2^_adjusted_	*B*	*SE B*	*95% CI*	β		*p*
**Extinction recall magnitude**
*US expectancies*							
STAIT	-0.021	-0.030	0.066	[-0.16, 0.10]	-0.030		
ACS Focusing	0.017	-0.259	0.135	[-0.53, 0.01]	-0.259		0.166
						*F*(2,42) = 1.87	
*SCR*							
STAIT	-0.060	0.002	0.005	[-0.009, 0.013]	0.101		
Orienting_ Costs	0.061	0.006	0.003	[0.000, 0.011]	0.475		0.149
						*F*(2,15) = 2.17	
*FPS*							
STAIT	-0.062	-0.201	0.442	[-1.16, 0.76]	-0.114		
Orienting_ Benefits	0.074	0.632	0.322	[-0.06, 1.32]	0.496		0.165
						*F*(2,12) = 2.10	
**Extinction learning gradient**
*SCR*							
STAIT	0.124	61.03	113.99	[-180.64, 302.69]	0.177		
Executive control	-0.003	32.99	33.91	[-38.89, 104.89]	0.321		0.138
						*F*(2,16) = 2.24	
*FPS*							
STAIT	0.041	-93.53	107.94	[-323.61, 136.54]	-0.209		
Orienting	0.059	-64.48	44.97	[-160.32, 31.37]	-0.346		0.177
						*F*(2,15) = 1.95	
STAIT	0.041	-176.90	90.94	[-370.73, 16.94]	-0.395		
Orienting_Costs	**0.274^∗^**	-100.15	36.82	[-178.63, -21.66]	-0.552		0.023
						*F*(2,15) = 4.91	
STAIT	0.041	-136.52	98.17	[-345.76, 72.72]	-0.305		
Alerting	0.142	67.43	34.63	[-6.39, 141.24]	0.427		0.086
						*F*(2,15) = 2.91	
**Extinction recall gradient**
*US expectancies*							
STAIT	-0.038	0.96	3.23	[-5.76, 7.67]	0.055		
Orienting	0.024	-2.66	1.37	[-5.48, 0.16]	-0.364		0.171
						*F*(2,25) = 1.90	
STAIT	-0.038	3.75	3.33	[-3.12, 10.61]	0.215		
Orienting_Benefits	**0.118^∗∗^**	-4.50	1.70	[-8.00, -0.99]	-0.505		0.046
						*F*(2,25) = 3.50	
*FPS*							
STAIT	-0.111	30.07	35.57	[-51.95, 112.08]	0.257		
Executive control	0.106	-21.54	9.86	[-44.28, 1.20]	-0.664		0.154
						*F*(2,8) = 2.39	


*US, unconditioned stimulus; SCR, skin conductance response; FPS, fear-potentiated startle; STAIT, State-Trait Anxiety Inventory, trait version. Significant values in bold*. ^∗^*p = 0.016*; ^∗∗^*p = 0.014*.

After controlling for trait anxiety, orienting benefits were also a significant predictor of extinction recall gradient (as measured by US expectancies), accounting for 12% of its variance (*F*[2,25] = 3.50, *p* = 0.046, *R^2^_Adjusted_* = 0.12). Thus, facilitated orientation by valid neutral cues predicted faster extinction recall beyond what is attributable to trait anxiety.

### Additional Analyses

When we calculated the gradient of fear extinction considering only the response to the CS+ (instead of the difference between CS+ and CS-) (Barry et al., 2017), we found that enhanced alerting was associated with faster extinction recall (as measured by FPS) (*r* = -0.695; *p* = 0.008) (see Supplementary Table S3).

## Discussion

In this paper, we found that individual differences in self-reported non-emotional AC are not significantly associated with fear extinction learning and recall. However, we did find that the two components of orienting network functioning are significantly negatively associated with fear extinction learning and recall beyond that accounted for by trait anxiety. Specifically, participants with enhanced orienting costs (i.e., difficulties in disengaging attention from cues not relevant for the task) showed faster extinction learning, while those with enhanced orienting benefits (i.e., facilitated orientation by valid cues) exhibited faster extinction recall as measured by FPS and US expectancies, respectively.

The lack of a significant association between self-reported non-emotional AC and fear extinction is at odds with the findings of Barry et al. (2016a), who reported that higher self-reported emotional AC was associated with faster fear extinction learning. In our calculation of the extinction gradients, we considered the difference between CS+ and CS- in each trial; however, when we used the same criteria as Barry and colleagues (Barry et al., 2016a,b) (i.e., considering only the response to the CS+) the results did not change (see Supplementary Table S3). The main difference between Barry’s studies and ours is that we measured AC in non-emotional conditions whereas Barry et al. (2016a, 2017) measured emotional AC. This underlines the importance of the distinction between emotional and non-emotional AC.

Our results on orienting network functioning are not easily comparable to previous research because, to our knowledge, this is first study to analyze how individual differences in the functioning of attentional networks under non-emotional conditions are associated with individual differences in fear extinction learning and recall. Our results on orienting costs resemble those reported of Waters and Kershaw (2015) using threat-related stimuli. While Waters and Kershaw (2015) found that higher *allocation to threat* was a predictor of *higher* extinction learning, we found that lower *capacity for disengaging attention from irrelevant cues* was a predictor of faster extinction learning. It may be that both processes tap into a similar construct that becomes apparent using both threat-related and non-emotional conditions. Consistent with this, Heeren et al. (2015b) stated that specific impairment of the orienting network, such as difficulty in disengaging attention from task-irrelevant distractors, may be a mechanism underlying attentional bias. Then, during fear extinction learning we could assume that people with greater orienting costs with non-emotional stimuli also display higher attentional allocation to the CS+ that speeds up the extinction learning process.

However, this interpretation conflicts with data showing that higher self-reported emotional AC is positively associated with faster extinction learning, as shown by Barry et al. (2016a). Nevertheless, in the study by Barry et al. (2017), a different stimulus was presented during extinction learning and during conditioning. The authors interpreted that participants with high AC were better able to shift attention from the common, threatening features of the original CS+ to the distinct features of a different but similar CS+, therefore “speeding up” the extinction learning process.

The fact that orienting costs and orienting benefits showed a different association with extinction learning and recall also suggests that distinct attentional capabilities may have different relationships with various fear learning processes. This is also consistent with previous studies showing that extinction learning and extinction recall are independent processes (Phelps et al., 2004; Milad et al., 2007; Quirk and Mueller, 2008).

In a previous study, Morillas-Romero et al. (2015) reported that, under non-emotional conditions, orienting network functioning was not related to spontaneous emotion regulation, but with explicit emotion regulation styles. To our knowledge, ours is the first report showing that individual differences in orienting network functioning under non-emotional conditions are related to a form of spontaneous emotion regulation (i.e., fear extinction). Our results underline the potential prominence of the orienting component of attention in anxiety and related processes, as shown recently by Heeren et al. (2015b), Heeren and McNally (2016) in adults with social anxiety disorder.

We also explored the contribution of the alerting network to fear extinction, and found that enhanced alerting was associated with faster fear extinction recall. Previous studies (Tortella-Feliu et al., 2014) have shown that the alerting network is related to self-reported emotion regulation strategies (i.e., enhanced alerting predicts a higher probability of suppressing distressing cognitions). Therefore, there seems to be a positive relationship between alertness and emotional regulation, and future research will need to investigate this relationship further.

Our results could have several methodological and clinical implications. From a methodological perspective, our data indicate that fear extinction gradients can provide a better understanding of individual differences in fear learning (and, more generally, a broader view of fear learning processes) than those offered by “static” fear indices, as previously observed by Barry et al. (2016a, 2017). They support that the empirical exploration of individual differences in non-emotional AC can be relevant for several clinical phenomena, as recently emphasized by Heeren et al. (2015b), and shown here for extinction learning and recall. Furthermore, our results highlight the utility of performance-based measures of attentional network functioning beyond general and self-reported AC.

Taken together with previous studies on the association between both fear extinction (e.g., Waters and Pine, 2016; Ball et al., 2017) and attentional functioning (e.g., Barry et al., 2015a) and exposure-based interventions, our results suggest that differences in the orienting network functioning may explain differences in exposure therapy outcomes, a question that deserves to be explored in future studies.

Our result may also have implications for other forms of psychological interventions, especially attention training. Attention training is a generic term that refers to repetitive “*practice in conflict-related tasks, working memory tasks or others tasks involving executive control mechanisms*” (Tang and Posner, 2009, p. 222) that requires directed effortful attention control. Importantly, most of these interventions train attention using non-emotional materials, as is the case of the attention training technique for anxiety disorders (Fergus and Bardeen, 2016; Knowles et al., 2016). However, in the field of anxiety disorders the most common attention intervention is attention bias modification (ABM) training (Bar-Haim, 2010). Positive results on the use of ABM, mainly as an add-on to cognitive behavior therapy, have been reported in several anxiety disorders (Linetzky et al., 2015). However, results on the efficacy of these interventions are inconsistent (Mogoaşe et al., 2014; Kuckertz and Amir, 2015), and some authors have called for ABM procedures to be improved (Mogg and Bradley, 2016). Notably, although these procedures are intended to train participants to disengage attention from threatening information, several studies have found that any active attentional training procedure will reduce anxiety symptoms, including procedures that use only neutral stimuli (Klumpp and Amir, 2010; Heeren et al., 2015c). Heeren et al. (2015c) also reported that several attention training procedures improve the executive control and alerting components of AC, but not the orienting one, in socially anxious patients. Therefore we propose that attentional training with neutral stimuli should be further explored as a way to improve AC, especially by directly manipulating the orienting network, as proposed by Heeren et al. (2015b). This in turn could be related to increased effectiveness of exposure.

Our study has several strengths. It is the first to explore the association between non-emotional AC and fear learning. Moreover, we measured fear learning using three different fear measures, controlled for some variables that are known to affect fear learning (menstrual cycle, time of the day), and used both magnitude and gradient-based measures. Also, our AC variables included self-reported and performance-based measures. We included analyses and results for the whole sample and only for those participants with successful conditioning and extinction learning. Finally, while previous studies focused on extinction learning, we also studied extinction recall.

We also note some limitations. Participants in our study were individuals with moderate to strong fear of spiders, which also involves disgust, not only fear (Cisler et al., 2009), and this may affect some of the processes investigated. Second, in the paradigm employed here conditioning and extinction learning occurred consecutively with no time for longer consolidation of fear conditioning. Third, some of our analyses including only participants with successful conditioning and extinction learning (those involving SCR and FPS measures and magnitude-based indices) were based on relatively small samples (less than a half of the whole sample for some analyses). However, the main findings from these analyses are fully consistent with those obtained using the whole sample (*n* = 50), which is a relatively large one for fear learning psychophysiological experiments using a 2-day procedure, compared to many previous studies (e.g., Milad et al., 2005; Rabinak et al., 2013, 2014; Garfinkel et al., 2014; Pineles et al., 2016). We computed post-hoc power (two-tailed) for our significant bivariate correlations using sample size, the effect size, and the alpha error (0.05). We had 53–63% (moderate) power to detect a significant correlation between the orienting network functioning and extinction learning and recall. This indicates that Type II error probability was still relatively high in our study, and therefore a replication with larger samples is warranted to ensure the generalizability of our findings. Finally, most significant findings could not be replicated across different fear measures, although this is consistent with many previous fear-learning studies (e.g., Sevenster et al., 2014).

Despite these limitations, we consider that these results contribute to a better understanding of how non-emotional AC is related to fear extinction learning and recall. The most important theoretical contribution is that attentional biases to threat in anxiety could reflect a much broader dysregulation of AC observed in face of non-emotional material.

In summary, in this exploratory study we showed that orienting network functioning is related to fear extinction learning and recall over and above trait anxiety. To the best of our knowledge, this is the first study that links non-emotional AC to fear extinction. An important avenue for future research is to explore the association between AC and anxiety treatments (i.e., exposure therapy).

## Ethics Statement

This study was carried out in accordance with the recommendations of Clinical Research Ethics Committee of Institut Hospital del Mar d’Investigacions Mèdiques, with written informed consent from all subjects. All subjects gave written informed consent in accordance with the Declaration of Helsinki. The protocol was approved by the Clinical Research Ethics Committee.

## Author Contributions

EF, DT-R, MF, and MT-F contributed to the development of the study hypothesis, and to the contribution of the study and experiment designs. EF, DT-R, and DT prepared the stimuli, and prepared data and software for analysis. EF, DT-R, and MT-F performed the data analysis. All authors drafted the manuscript, discussed the results, implications, and literature, and approved the final version of the manuscript for submission.

## Conflict of Interest Statement

The authors declare that the research was conducted in the absence of any commercial or financial relationships that could be construed as a potential conflict of interest.
