# Supplementary material for: Attentional Control and Fear Extinction in Subclinical Fear: An Exploratory Study
Source: Front Psychol. 2017 Sep 26;8:1654. doi: 10.3389/fpsyg.2017.01654 (PMC5622961; doi:10.3389/fpsyg.2017.01654)
Supplement: Supplementary file 1 [file Data_Sheet_1.docx]

**Attentional Control and Fear Extinction in Subclinical Fear: an Exploratory Study**

Forcadell et al.

**SUPPLEMENTARY MATERIALS**

**Methods and Results**

## Methods

**Attentional Network Functioning**

Following standard criteria (Callejas, Lupiáñez, & Tudela, 2004; Pacheco-Unguetti, Acosta, Callejas, & Lupiáñez, 2010; Pacheco-Unguetti, Acosta, Marqués, & Lupiáñez, 2011), we computed an efficiency index for each attentional network: executive control = incongruent–congruent trials; orienting = invalid–valid trials; alerting = no alerting–alerting tone (restricted to the uncued condition). Orienting was also divided into costs (i.e. difficulties in disengaging attention from invalid cues) = invalid–uncued trials, and benefits (i.e. facilitated orientation) = uncued–valid trials.

## Fear learning paradigm

We adapted the 2-day fear learning paradigm developed by Milad, Orr, Pitman, & Rauch (2005).

The US (electric shock) had a duration of 100 ms and was generated by a Grass S48 stimulator, transmitted via a constant current unit, and delivered to the volar surface of the non-dominant forearm using a bipolar bar electrode. It was adjusted for each participant to be ‘definitely annoying but not painful’. Participants were told that they would see images of two different rooms during the experimental task and that they may or may not be shocked. They were also told that, in case they were shocked, it would be at the end of the presentation of the images.

## Before the experimental task, participants received four practice trials (in which the two CSs and the two contexts were combined). Then six startle probes were presented, in order to further habituate responding.

The procedure on the first part of day 2 was the same as on day 1, with the following exceptions: participants were told that the shock intensity would be the same as that selected the day before, they were reminded of the instructions, and were told that they had to use their memory of what they had learned the previous day to predict the occurrence of the US (shock). They did not receive any practice trials, and 10 habituation startle probes were presented before the extinction recall.

**Recording and quantification**

**Skin conductance response (SCR)**

Physiological measures were recorded using a Biopac 150 polygraph (Biopac Systems, Inc). SCR was recorded at the distal phalanges of the index and middle fingers of the non-dominant hand using two Ag-AgCl electrodes filled with electrolyte. The signal was sampled at a rate of 125 Hz.

**Fear-potentiated startle (FPS)**

The startle blink was measured by recording the electromyographic activity (EMG) of the orbicularis oculi, using two 0.5 cm Ag-AgCl surface electrodes. The raw EMG signal was sampled at a rate of 2000 Hz, filtered (analogue 50-Hz notch filter; and digital infinite-impulse-response, 28 to 500 Hz, band-pass filter), and rectified and smoothed offline (10-ms moving window average).

**Results**

**Preliminary analyses:**

**ANT-I reaction time analysis**

We examined reaction time in the ANT-I using a factorial mixed ANOVA for overall intra-subject effects. In line with previous studies (e.g. Pacheco-Unguetti et al., 2011), extreme values (faster than 200 and slower than 1200 ms) were eliminated to avoid anticipation and very long response latencies, respectively. Each of the 50 participants performed a total of 192 trials in the ANT-I task, for a total of 9600 trials (50 × 192). Reaction time for response trials as a dependent variable was introduced into a 2 (executive control: congruent, incongruent) x 3 (orienting: valid, invalid, uncued) x 2 (alerting: no alerting, alerting tone) factorial mixed ANOVA to explore overall attentional effects. After eliminating extreme values, mean reaction time per experimental condition and error rates are depicted in Table S1.

Consistent with previous studies (Callejas et al., 2004; Pacheco-Unguetti et al., 2010, 2011), we observed significant main effects for executive control (*F*[1, 49]=488.7, *p*< .001, η^2^ = .909, mean square error (*MSE*) *= 1167.56,*= 1961.8), orienting (*F*[2, 98] = 157.4, *p*< .001, η^2^ = .763, *MSE* = 697.6) and alerting (*F*[1, 49] = 101.9, *p*< .001, η*^2^*= .675, *MSE* = 1182,8). Specifically, responses were significantly faster in trials where distracters pointed in the same direction as the arrow target (i.e. congruent trials) compared to those where distracters pointed in the opposite direction, in trials with an orienting signal compared to those without, and in trials with an alerting tone compared to those without.

We also observed significant interactions between attentional networks. The interaction between executive control and orienting (*F*[2, 98] = 12.87, *p*< .001, η^2^= .208, *MSE* = 546.6) showed a reduction in the congruency effect in valid trials. In the interaction between executive control and alerting (*F*[1, 49] = 26.9, *p*< .001, η^2^ = .355, *MSE* = 484.5), we observed a larger congruency effect when the alerting tone was presented. Finally, in the orienting and alerting interaction (*F*[2, 98] = 47.43, *p*< .001, η^2^ = .492, *MSE* = 436.8), we found longer reaction time differences between cued and uncued trials with the alerting tone. All these main effects and interactions were consistent with the pattern usually observed for this task (e.g.Callejas et al., 2004; Pacheco-Unguetti et al., 2010, 2011).

**Evidence of conditioning and fear extinction**

We found evidence of successful conditioning (i.e. higher response to the CS+ than to the CS- in the last block of conditioning) for all measures (US expectancies, SCR, and FPS). We also found evidence of successful extinction learning (i.e. similar response to the CS+ and CS- in the last block of extinction) for all measures (US expectancies, SCR, and FPS). Finally, we only observed successful extinction recall (i.e. similar response to the CS+ and CS- in the extinction context during the first block of extinction recall) for FPS. See Forcadell et al., 2017 for further information.

**Additional analyses:**

**Relationships between trait anxiety and attentional variables**

Since trait anxiety has been widely reported to be related to lower attentional control, we also examined the relationships between these variables. We found that trait anxiety was negatively associated with self-reported attentional control (*r* = -.404, *p* =.004 for the overall scale; *r* = -.393, *p* = .005 for the focusing subscale, and *r* = -.303, *p* = .032 for the shifting subscale) and performance-based attentional control (i.e. greater interference in the executive control network, *r* = .279, *p* = .049). No significant associations were found for the orienting (*p* = .415) and alerting (*p* = .961) networks.

**Supplementary references**

Callejas, A., Lupiáñez, J., & Tudela, P. (2004). The three attentional networks: On their independence and interactions. *Brain and Cognition*, *54*(3), 225–227. doi: 10.1016/j.bandc.2004.02.012

Dunsmoor, J. E., Mitroff, S. R., & LaBar, K. S. (2009). Generalization of conditioned fear along a dimension of increasing fear intensity. *Learning & Memory*, *16*(7), 460–9. doi: 10.1101/lm.1431609

Forcadell, E., Torrents-Rodas, D., Vervliet, B., Leiva, D., Tortella-Feliu, M., and Fullana, M. A. (2017). Does fear extinction in the laboratory predict outcomes of exposure therapy? A treatment analog study. *Int. J. Psychophysiol*. Advance online publication. doi: 10.1016/j.ijpsycho.2017.09.001

Milad, M. R., Orr, S. P., Pitman, R. K., & Rauch, S. L. (2005). Context modulation of memory for fear extinction in humans. *Psychophysiology*, *42*(4), 456–464. doi: 10.1111/j.1469-8986.2005.00302.x

Pacheco-Unguetti, A. P., Acosta, A., Callejas, A., & Lupiáñez, J. (2010). Attention and anxiety: different attentional functioning under state and trait anxiety. *Psychological Science*, *21*(2), 298–304. doi: 10.1177/0956797609359624

Pacheco-Unguetti, A. P., Acosta, A., Marqués, E., & Lupiáñez, J. (2011). Alterations of the attentional networks in patients with anxiety disorders. *Journal of Anxiety Disorders*, *25*(7), 888–895. doi: 10.1016/j.janxdis.2011.04.010

**Extinction learning gradient_ FPS**

**Orienting costs**

**Orienting benefits**

**Extinction recall gradient_US expectancies**

*Figure S1.*Scatter plots for the main significant correlations.

a) Scatter plot for the correlation between extinction learning gradient (as measured by FPS) and orienting costs (when only those participants with successful conditioning and extinction learning were included). Results remained unchanged when one possible outlier was excluded (a participant with a score 3 standard deviations above the mean).

b) Scatter plot for the correlation between extinction recall gradient (as measured by US expectancies) and orienting benefits (results for the whole sample). Results remained unchanged when one possible outlier was excluded (a participant with a score 3 standard deviations below the mean).

FPS: Fear-Potentiated startle; US: Unconditioned stimulus.

Table S1.

*Mean reaction times (in milliseconds) and error rates (in parentheses) for each condition of the Attentional Network Test for Interactions.*

|  | Without alerting tone | | | | With alerting tone | | |
| --- | --- | --- | --- | --- | --- | --- | --- |
|  | Neutral | Valid | Invalid | Neutral | | Valid | Invalid |
| Congruent | 565(.01) | 519(.00) | 552(.01) | 502(.00) | | 491(.00) | 530(.00) |
| Incongruent | 626(.02) | 590(.03) | 632(.04) | 590(.02) | | 565(.02) | 637(.06) |

Table S2.

*Bivariate correlation between self-reported attentional control and attentional network functioning in the Attentional Network Test for Interaction (n = 50)*

|  | ACS total | ACS focusing | ACS shifting |
| --- | --- | --- | --- |
| Executive control | **-,438**  (*p* = .001) | **,323**  (*p* = .022) | ,106  (*p* = .464) |
| Orienting | -,136  (*p* =,345) | ,124  (*p* =,391) | ,160  (*p* =,268) |
| Orienting-costs | ,085  (*p* =.556) | -,087  (*p* =.549) | ,069  (*p* =.633) |
| Orienting-benefits | -,243  (*p* =.088) | ,230  (*p* =.108) | ,122  (*p* =.401) |
| Alerting | -,057  (*p* =.694) | -,162  (*p*=.260) | -,245  (*p*=.087) |

ACS: Attentional Control Scale. Significant values in bold.

Table S3.

*Bivariate correlation between attentional control and fear extinction gradient-based indices (considering responses to CS+).*

|  | AC TOTAL | AC focusing | AC shifting | Executive control | Orienting | Orienting-costs | Orienting-benefits | Alerting |
| --- | --- | --- | --- | --- | --- | --- | --- | --- |
| EXTINCTION LEARNING GRADIENT |  |  |  |  |  |  |  |  |
| US expectancies n=46 | .073  (*p*=.631) | .059  (*p*=.695) | .035  (*p*=.815) | .015  (*p*=.919) | .010  (*p*=.946) | -.113  (*p*=.456) | .119  (*p*=.413) | .001  (*p*=.994) |
| SCR n=18 | -.297  (*p*=.232) | -.307  (*p*=.216) | -.219  (*p*=.383) | .100  (*p*=.693) | -.004  (*p*=.987) | .199  (*p*=.428) | -.176  (*p*=.484) | -.169  (*p*=.504) |
| FPS n=22 | .212  (*p*=.344) | .003  (*p*=.990) | .295  (*p*=.183) | .003  (*p*=.988) | .050  (*p*=.824) | .141  (*p*=.533) | -.085  (*p*=.708) | .277  (*p*=.213) |
| EXTINCTION RECALL GRADIENT |  |  |  |  |  |  |  |  |
| US expectancies n=32 | -.037  (*p*=.839) | .007  (*p*=.971) | -.076  (*p*=.679) | .092  (*p*=.617) | .001  (*p*=.997) | -.131  (*p*=.473) | .121  (*p*=.510) | .163  (*p*=.373) |
| SCR n=17 | .246  (*p*=.342) | .002  (*p*=.994) | .310  (*p*=.226) | -.305  (*p*=.233) | .404  (*p*=.107) | .389  (*p*=.122) | .178  (*p*=.494) | -.281  (*p*=.274) |
| FPS n=13 | .017  (*p*=.955) | .115  (*p*=.708) | -.156  (*p*=.610) | -.078  (*p*=.801) | -.030  (*p*=.921) | .180  (*p*=.556) | -.365  (*p*=.220) | **-.695**  **(*p*=.008)** |

CS+, conditioned stimulus associated with the unconditioned stimulus during conditioning; US, unconditioned stimulus; SCR, skin conductance response; FPS, fear-potentiated startle. Significant values in bold.
